# Supplementary material for: Computational prediction of ω-transaminase selectivity by deep learning analysis of molecular dynamics trajectories
Source: QRB Discov. 2022 Dec 12;4:e1. doi: 10.1017/qrd.2022.22 (PMC10392675; doi:10.1017/qrd.2022.22)
Supplement: Supplementary file 1 [file S2633289222000229sup001.pdf]

Supporting Information

Computational prediction of ω-transaminase selectivity by deep learning analysis of molecular dynamics trajectories

Carlos Ramírez-Palacios[1] and Siewert J. Marrink[1]*

[1] Molecular Dynamics, Groningen Biomolecular Sciences and Biotechnology Institute (GBB), University of Groningen, Nijenborgh 7, 9747 AG Groningen, Groningen, The Netherlands

* Corresponding author
Groningen Biomolecular Sciences and Biotechnology Institute
University of Groningen
Nijenborgh 7
9747 AG Groningen
The Netherlands
E-mail: s.j.marrink@rug.nl

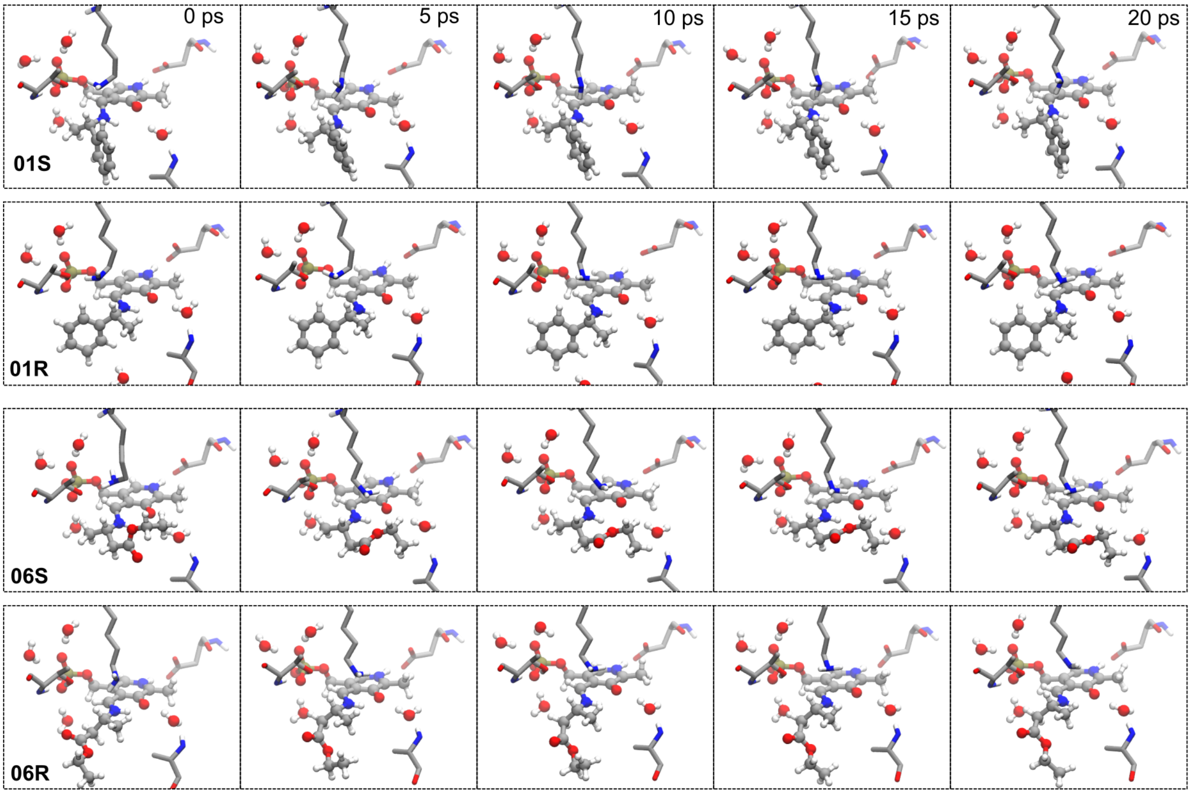

Figure S1. Dynamics of the studied complexes during the 20 ps MD simulations. The trajectories sample local regions of the conformational space of the docked complexes. We were unable to distinguish by visual inspection between simulations of the "non-reactive" (e.g., **01R** and **06R**) and simulations of the "reactive" (e.g., **01S** and **06S**) enantiomers. Conversely, the NNs were able to tell the two classes apart using these trajectories.

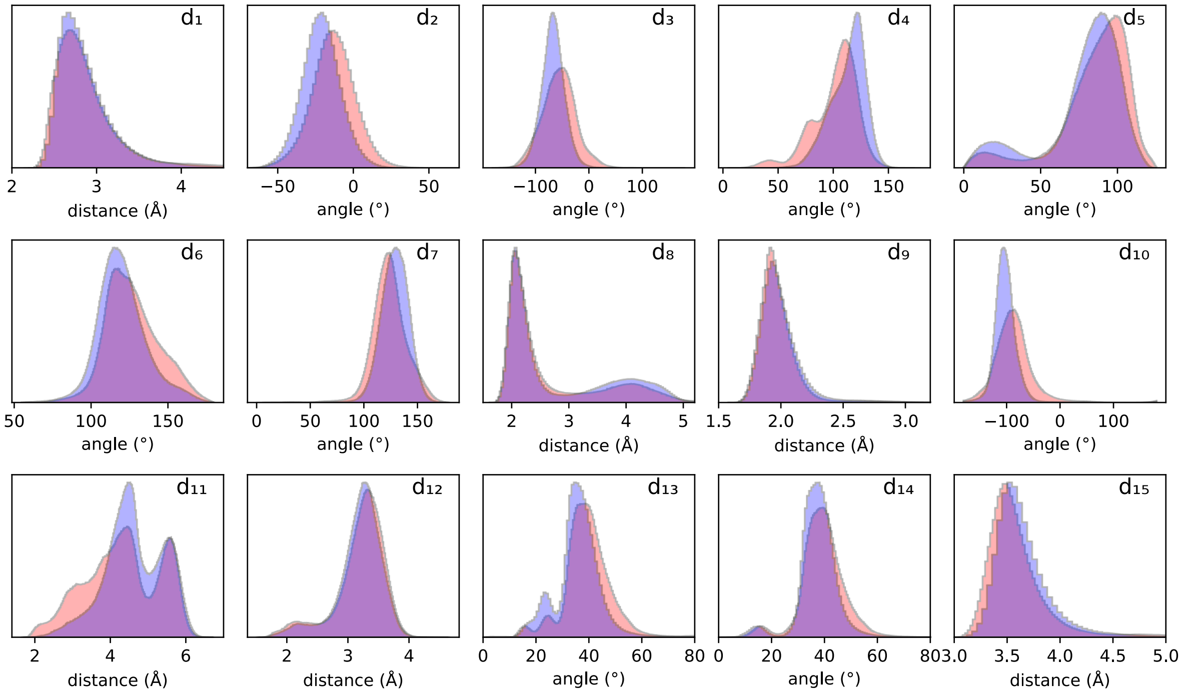

Figure S2. Distribution of the fifteen descriptors in all 9,800 trajectories. Colors are *red* for the "non-reactive" class, and *blue* for the "reactive" class.

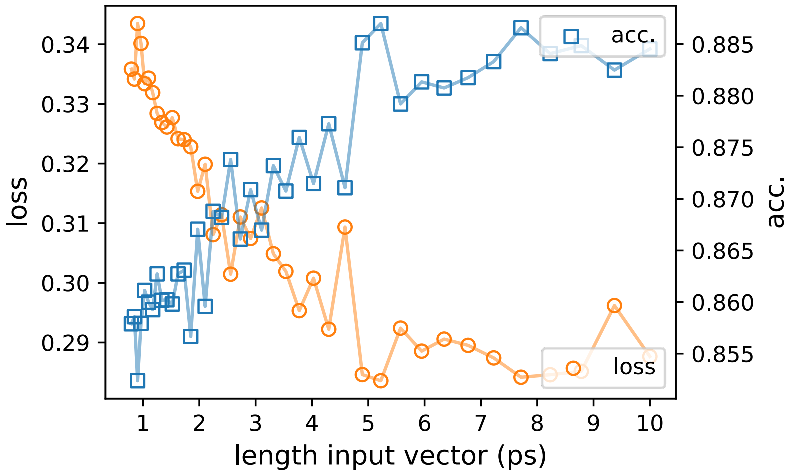

Figure S3. Performance of the 1D-CNN model (*y-axis*) upon changing the length of the input vectors (*x-axis*), in the range 0.8 – 10 ps. Trajectories shorter than the standard 20 ps (e.g., 5 – 10 ps) were long enough for a trained network to achieve good performance. Trajectories shorter than 5 ps did exhibit a reduced performance, but binary classification remained possible (acc. >> 0.500) even with sub-ps trajectories. Losses and accuracies were averaged over 10 replicas.

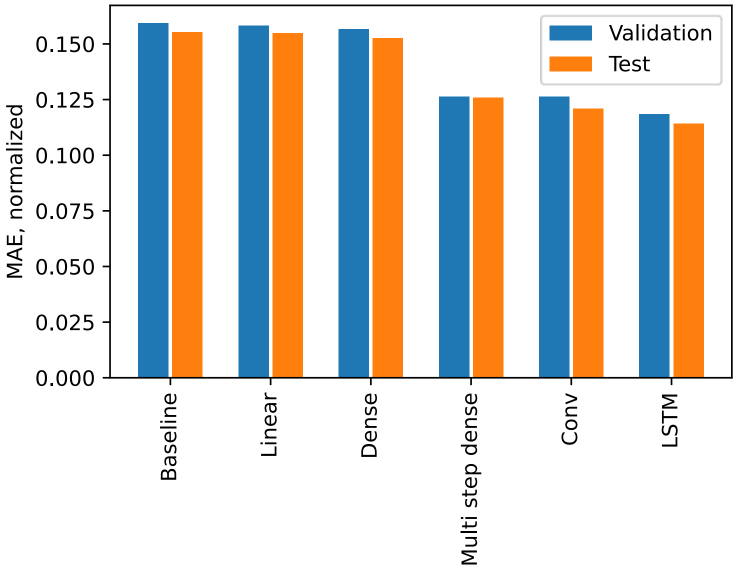

Figure S4. Performance of various models in the time-series forecasting problem. The LSTM model achieved slightly better performance than all the other models shown as reference. The hyperparameters for the baseline, linear, dense, multi-step dense, and convolutional models were the same as the ones employed in the Keras time-series forecasting tutorial (https://www.tensorflow.org/tutorials/structured_data/time_series).
